# Patient-Centered Research Through Artificial Intelligence to Identify Priorities in Cancer Care

**DOI:** 10.1001/jamaoncol.2025.0694

**Published:** 2025-04-24

**Authors:** Jiyeong Kim, Michael L. Chen, Shawheen J. Rezaei, Mariana Ramirez-Posada, Jennifer L. Caswell-Jin, Allison W. Kurian, Fauzia Riaz, Kavita Y. Sarin, Jean Y. Tang, Steven M. Asch, Eleni Linos

**Affiliations:** 1 Center for Digital Health, Stanford University School of Medicine, Stanford, California; 2 Department of Dermatology, Stanford University School of Medicine, Stanford, California; 3 Division of Oncology, Department of Medicine, Stanford University School of Medicine, Stanford, California; 4 Department of Epidemiology and Population Health, Stanford University School of Medicine, Stanford, California; 5 Division of Primary Care and Population Health, Stanford University School of Medicine, Stanford, California

## Abstract

**Importance:**

Patient-centered research is essential for bridging the gap between research and patient care, yet patient perspectives are often inadequately represented in health research.

**Objective:**

To leverage artificial intelligence (AI) and natural language processing (NLP) to analyze a large dataset of patient messages, defining patient concerns and generating relevant research topics, and to quantify the quality of these AI-generated topics.

**Design, Setting, and Participants:**

This case series was conducted using an automated framework involving a 2-staged unsupervised NLP topic model and AI-generated research topic suggestions. The study was based on deidentified patient portal message data from individuals with breast or skin cancer at Stanford Health Care and 22 affiliated centers over July 2013 to April 2024.

**Exposures:**

A widely used large language model (ChatGPT-4o [OpenAI]; April 2024) was used and guided through multiple prompt-engineering strategies to perform multilevel tasks, including knowledge interpretation and summarization (eg, interpreting and summarizing the NLP-defined topics), knowledge generation (eg, generating research ideas corresponding to patients’ issues), self-reflection and correction (eg, ensuring and revising the research ideas after searching for scientific articles), and self-reassurance (eg, confirming and finalizing the research ideas).

**Main Outcomes and Measures:**

Three breast oncologists (J.L.C., A.W.K., F.R) and 3 dermatologists (K.Y.S, J.Y.T., E.L.) evaluated the meaningfulness and novelty of the AI-generated research topics using a 5-point Likert scale (1 representing exceptional to 5 representing poor). Mean (SD) scores for meaningfulness and novelty were computed for each topic.

**Results:**

A total of 614 464 patient messages were analyzed from 25 549 individuals, 10 665 with breast cancer (98.6% female) and 14 884 had skin cancer (49.0% female). The overall mean (SD) scores for meaningfulness and novelty were 3.00 (0.50) and 3.29 (0.74), respectively, for breast cancer topics and 2.67 (0.45) and 3.09 (0.68), respectively, for skin cancer topics. One-third of the AI-suggested research topics were highly meaningful and novel when both scores were lower than the average (5 of 15 for breast cancer and 6 of 15 for skin cancer). Notably, two-thirds of the AI-suggested topics were novel (10 of 15 for breast cancer and 11 of 15 for skin cancer).

**Conclusions and Relevance:**

This case series demonstrates that AI/NLP-driven analysis of large volumes of patient messages can generate quality research topics in cancer care that reflect patient perspectives, providing valuable guidance for future patient-centered health research endeavors.

## Key Points

**Question:**

Can artificial intelligence (AI) and natural language processing generate quality research ideas that prioritize patient concerns by analyzing a large database of patient portal messages?

**Findings:**

In this case series of 614 464 messages from 25 549 patients with breast or skin cancer, AI-generated research topics were evaluated by 6 highly experienced clinicians for meaningfulness and novelty. One-third of topics were highly meaningful and novel, and two-thirds were novel.

**Meaning:**

AI/natural language processing–enabled analysis of patient messages can generate valuable research topics that prioritize patient perspectives in cancer care, guiding future patient-centered health research.

## Teaser

This case series uses artificial intelligence and natural language processing to analyze a large dataset of patient messages to inform patient-centered research, counseling, and quality improvement initiatives in cancer care.

## Introduction

Patient-centered research is critical for bridging the gap between research and patient care, ensuring clinically meaningful outcomes.^1^ Despite long-standing efforts to include patient perspectives in health research, traditional methods face considerable barriers, including high resource demands and inconsistent patient engagement.^2,3^ Over the past decade, patient portal messaging within electronic health record (EHR) systems has become a primary means for patients to communicate their clinical concerns to clinicians.^4^ The COVID-19 pandemic has further amplified the use of these EHR-based communications, doubling the volume.^5,6^

Collecting extensive patient input is challenging and resource intensive, but patient portal messages offer a valuable and underused resource for real-time identification of patient concerns. Natural language processing (NLP), a probability-based language model, can extract key information from vast text datasets.^7,8,9,10^ Advances in artificial intelligence (AI) have propelled the use of AI-based NLP in research, enabling the analysis of large volumes of patient-generated data, such as social media and patient forums.^11,12,13,14^ Large language models (LLMs) have demonstrated impressive performance in various research tasks, including providing peer-review article feedback and assisting in patient selection for clinical trials.^15,16^

Using a large database of secure messages from patients with cancer, we leveraged a framework of AI-enabled NLP to prioritize patient-discussed issues and generate patient-centered research topics. We further validated these topics with domain experts. This pilot case series demonstrates how AI-enhanced NLP can prioritize patient concerns, thereby informing patient-centered research, counseling, and quality improvement initiatives in cancer care.

## Methods

### Data Source and Study Design

We obtained deidentified patient portal messages from individuals with breast or skin cancer (melanoma, basal cell carcinoma, or squamous cell carcinoma) defined using *International Statistical Classification of Diseases and Related Health Problems, Tenth Revision,* codes from Stanford Health Care and 22 affiliated centers over July 2013 to April 2024. Only messages labeled as a patient medical advice request (PMAR) routed to oncology or dermatology were included. Patient characteristics were also collected from the EHR system, including sex and race and ethnicity (Asian, Black, Hispanic, Native American or Pacific Islander, White, race other than those listed, and unknown). Race and ethnicity were reported to present the distribution of patient demographics.

The institutional review board at Stanford University approved the study and granted waiver of informed consent owing to retrospective use of deidentified data. We followed Strengthening the Reporting of Observational Studies in Epidemiology (STROBE) reporting guidelines.

### Two-Staged Topic Modeling

To identify patients’ clinical concerns, we analyzed the secure messages using a 2-staged unsupervised NLP topic model. This process used BERT (bidirectional encoder representations from transformers) and BIRCH (balanced iterative reducing and clustering using hierarchies) techniques.^17,18^ Preprocessed messages were converted into sentences with precalculated embedding and simplified.^19^ Similar topics were categorized via zero-shot clustering using cosine similarity scores. We further refined these clusters with a new BERTopic model, applying BIRCH algorithm, principal component analysis, and incremental fitting techniques for process efficiency (eMethods 1 in Supplement 1).^20^ This analysis highlighted the top 5 clinical concerns systematically selected based on exclusion criteria for both breast and skin cancer groups, excluding administrative issues (eTable 1 in Supplement 1).

### AI to Generate Research Topics

Using an LLM (ChatGPT-4o [OpenAI]; April 2024), we generated research topics to address patient concerns. We used multiple prompt-engineering strategies to provide contextual background, role prompting, directive commanding, expertise emulation, and zero-shot chain of thought.^21,22^ The AI performed multilevel tasks, including knowledge interpretation and summarization (eg, interpreting NLP-defined topics regarding patients’ clinical issues), generation (eg, generating research ideas corresponding to patients’ issues), self-reflection (eg, reflecting suggestions), and self-reassurance (eg, reassuring the meaningfulness and novelty after the article search) (eMethods 2 and eTable 2 in Supplement 1).

### Evaluation of AI-Generated Research Topics

Three breast oncologists (J.L.C., A.W.K., F.R) and 3 dermatologists (K.Y.S, J.Y.T., E.L.) assessed their agreement if LLM-interpreted topics were representative of patient concerns using a 5-point Likert scale (1 representing agree to 5 representing disagree) and the meaningfulness and novelty of the AI-generated research topics using a 5-point Likert scale (1 representing exceptional to 5 representing poor), adapted from grant-review scoring. These experts had between 10 and 30 years of clinical practice and extensive research experience. The meaningfulness and novelty scores were averaged, and standard deviations were calculated to gauge assessor agreement.^23^ Additionally, 2 researchers (S.J.R. and M.R.P.) independently conducted literature searches to confirm the novelty, and a third researcher (J.K.) confirmed. The search concluded if the terms ceased to appear relevant or on reaching the tenth page of Google Scholar (eTable 3 in Supplement 1).

### Statistical Analysis

We assessed the correlation between the 2 novelty measures using Spearman rank correlation test. AI-drafted research topics and their evaluations are summarized in eTable 4 in Supplement 1. We computed 2-sided *P *values and determined the statistical significance at *P* < .05 using Python, version 3.10 (Python Software Foundation).

## Results

### Study Population Characteristics

Among the 25 549 patients analyzed (10 665 with breast cancer and 14 884 with skin cancer), 11.7% were Asian, 1.0% were Black, 0.7% were Native American or Pacific Islander, 77.2% were White, 6.8% were a race other than those listed, and 2.6% were unknown, with 5.0% being of Hispanic ethnicity. Patients with breast cancer were predominantly White (61.1%) and female (98.6%), while patients with skin cancer were largely White (88.7%) with a balanced sex distribution (51.0% male and 49.0% female) (eTable 5 in Supplement 1).

### Patient Message Characteristics

A total of 44 984 615 unique message threads from 2013 to 2024 were identified across 14 672 401 patients (PMAR, 32.6%). This study focused on 614 464 PMARs: 474 194 from patients with breast cancer and 140 270 from patients with skin cancer (Figure).

**Figure.**
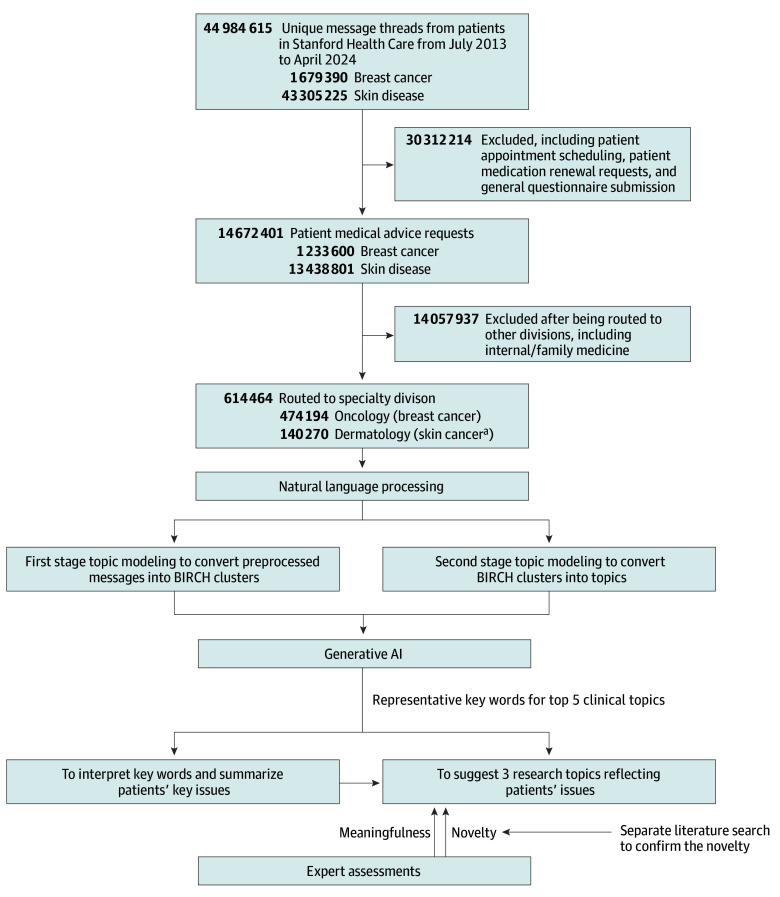
Data Source and Study Design AI indicates artificial intelligence; BIRCH, balanced iterative reducing and clustering using hierarchies. ^a^Skin cancer includes melanoma and other malignant neoplasms of the skin, including basal cell carcinoma and squamous cell carcinoma.

### Patients’ Clinical Concerns

The Box presents the primary clinical concerns of patients with cancer interpreted by AI using NLP-generated key words. Domain experts mostly agreed with LLM’s interpretation of patient concerns (breast cancer: mean [SD], 1.00 [0.00]; skin cancer: mean [SD], 1.33 [0.94]).

Box. **Primary Clinical Concerns of Patients With Breast or Skin Cancer Addressed Through Patient Portal Messages in 2013-2024^a^**

**For Patients With Breast Cancer**

**Topic 1. Key words:** *rash*, *itching*, *topical*, *allergic*, *lotion*, *redness*
  **Concerns:** Skin-related issues (eg, rashes and itching). These symptoms suggest possible allergic reactions or adverse effects from treatments, necessitating the use of topical treatments or lotions for alleviation.
**Topic 2. Key words:** *urine*, *urinalysis*, *urinating*, *bladder*, *peeing*, *cystitis*
  **Concerns:** Issues related to urinary function, including frequent urination, urinary tract infections, and bladder discomfort. Concerns include symptoms of cystitis and the need for urinalysis to diagnose potential problems. These issues may be exacerbated by treatment adverse effects or infections.
**Topic 3. Key words:** *dentist*, *tooth*, *periodontist*, *oral*, *filling*, *endodontist*
  **Concerns:** Dental health issues (eg, toothaches, dental procedures, oral hygiene). Messages mention the need for visits to the dentist, periodontist, or endodontist for treatments (eg, fillings and other procedures). These concerns highlight the importance of addressing oral health as part of the overall cancer care plan.
**Topic 4. Key words:** *genetic*, *geneticist*, *BRCA*, *testing*, *genomic*
  **Concerns:** Concerned about genetic testing, particularly *BRCA* gene alterations, and the implications for diagnosis and treatment. Patients seek guidance from geneticists on the necessity and benefits of undergoing genetic testing and understanding the results, including *BRCA2* alterations.
**Topic 5. Key words:** *liver*, *hepatic*, *ascites*, *biopsy*, *worried*, *ultrasound*
  **Concerns:** Liver-related issues (eg, hepatic conditions and ascites). Patients are worried about biopsy and ultrasonography results. Patients are anxious about liver health and its implications on their overall cancer management.

**For Patients With Skin Cancer**

**Topic 1. Key words:** *nasal*, *pimple*, *nostril*, *face*, *biopsy*, *surgery*, *skin*, *picture*
  **Concerns:** Issues about lesions or pimples on the nose and nostrils, which may resemble common skin issues but raise fears of malignant neoplasms. Patients often seek clarification on whether these lesions require a biopsy or surgery, particularly after sharing photographs with their clinician for evaluation.
**Topic 2. Key words:** *mole*, *melanoma*, *concerned*, *removal*, *sure*, *skin*, *checked*
  **Concerns:** Issues about the potential for moles to develop into melanoma. Patients express anxiety over whether certain moles should be removed or further examined and seek reassurance or confirmation through appointments. The need for timely skin checks and possible mole removal is a considerable focus of their concern.
**Topic 3. Key words:** *ear*, *earlobe*, *earrings*, *hearing*, *surgery*, *biopsy*, *cartilage*, *canal*
  **Concerns:** Issues about earlobes in relation to surgical interventions like biopsies and the impact on hearing. Patients are also worried about complications involving the cartilage and ear canal following surgeries or procedures. Additionally, patients are concerned about wearing earrings and the overall appearance of the ear after surgery.
**Topic 4. Key words:** *effudex*,^b^ *treatment*, *week*, *biopsy*
  **Concerns:** Issues with the treatment process involving Efudex (fluorouracil), focusing on its application duration (typically over several weeks), its effectiveness, and particularly, treatment impact following a biopsy.
**Topic 5. Key words:** *stitches*, *incision*, *sutures*, *surgery*, *wound*, *healing*
  **Concerns:** Issues about the management of surgical wounds related to stitches and sutures. Patient messages reflect worries about incision care, the healing process, and potential complications with stitching after surgery. They seek guidance on proper wound care and expect timely advice on how to manage it.

^a^
The top 5 clinical issues were included in the study, excluding issues that required administrative support, including scheduling (eg, appointments for radiation, chemotherapy, or infusion), authorization (eg, billing or payment), and paperwork (eg, Family and Medical Leave Act form). The artificial intelligence model generated 3 research topics for each clinical concern. Listed concerns are the large language model’s interpretation using key words extracted by the natural language processing model from patient messages. Key words represent the most important and frequent issues of patient messages.

^b^
Key words captured that the chemotherapy medication Efudex is commonly misspelled in patient messages (eg, Effudex or Efudix).

### Assessment of AI-Generated Research Topics

The overall meaningfulness score was lower than the novelty score (lower score means higher quality) in both breast cancer (meaningfulness: mean [SD], 3.00 [0.50]; novelty: mean [SD], 3.29 [0.74]) and skin cancer (meaningfulness: mean [SD], 2.67 [0.45]; novelty: mean [SD], 3.09 [0.68]) (Table). For breast cancer, meaningful and novel topics (when score was lower than mean) included: (1) interdisciplinary approach to managing dental health in breast cancer care (meaningfulness: mean [SD] score, 2.33 [1.15]; novelty: mean [SD] score, 2.33 [0.58]) and (2) evaluating the efficacy of hepatoprotective agents in preventing liver damage during breast cancer treatment (meaningfulness: mean [SD] score, 2.33 [1.15]; novelty: mean [SD] score, 3.00 [1.00]). For skin cancer, topics included (1) development and evaluation of a patient-centered digital tool for postsurgical wound care (meaningfulness: mean [SD] score, 2.33 [1.15]; novelty: mean [SD] score, 2.33 [0.58]) and (2) impact of patient education on Efudex (fluorouracil) treatment adherence and outcomes (meaningfulness: mean [SD] score, 1.67 [0.58]; novelty: mean [SD] score, 2.33 [1.53]).

**Table.** High-Quality Artificial Intelligence (AI)–Generated Research Topics Reflecting Patients’ Concerns

AI-generated research topics	Score, mean (SD)^a^	
	Meaningfulness	Novelty
**For patients with breast cancer**		
Interdisciplinary approach to managing dental health in breast cancer care	2.33 (1.15)	2.33 (0.58)
Evaluating the efficacy of hepatoprotective agents in preventing liver damage during breast cancer treatment	2.33 (1.53)	3.00 (1.00)
Longitudinal study on the impact of genomic testing on treatment outcomes	2.67 (1.15)	3.00 (1.00)
Development and testing of a specialized skin care regimen for patients with breast cancer	2.67 (1.15)	3.00 (1.00)
Efficacy of preventive dental care protocols for patients with breast cancer	3.00 (1.00)	2.33 (0.58)
**For patients with skin cancer**		
Development and evaluation of a patient-centered digital tool for postsurgical wound care	2.00 (0.00)	1.67 (0.58)
Impact of patient education on Efudex (fluorouracil) treatment adherence and outcomes	1.67 (0.58)	2.33 (1.53)
Longitudinal study on patient anxiety and decision-making in mole surveillance and removal	2.33 (1.53)	2.67 (1.53)
Impact of ear and earlobe reconstruction on hearing after skin cancer surgery	2.33 (0.58)	3.00 (1.73)
Impact of suture materials on scar formation in patients with skin cancer	2.67 (1.15)	2.67 (0.58)
Investigation of cartilage-sparing techniques in skin cancer surgery of the ear	2.67 (1.15)	2.67 (1.15)

^a^ Mean (SD) scores were computed based on the scores of 3 experts (oncologists J.L.C., A.W.K., and F.R or dermatologists K.Y.S, J.Y.T., and E.L.). Among 30 AI-generated research topics (15 in breast cancer and 15 in skin cancer), only high-quality topics were listed in this table in order of quality (from high to low) when both scores were better than the overall mean (a lower score means better quality). A full list of AI-generated research topics with experts’ scores is available in eTable 4 in Supplement 1.

One-third of the AI-suggested research topics were highly meaningful and novel when both scores were lower than the average (5 of 15 for breast cancer and 6 of 15 for skin cancer). Two-thirds of AI-generated topics were novel (10 of 15 for breast cancer and 11 of 15 for skin cancer). Expert novelty scores had a positive correlation with the absence of existing literature, though not conclusively (ρ, 0.28; 95% CI, −0.37 to 0.36; *P* = .13).

## Discussion

This study evaluated an LLM’s capability to generate patient-centered research topics and assessed the meaningfulness and novelty of these AI-generated topics. Approximately one-third of the proposed research topics were highly meaningful and novel, and two-thirds were novel for both breast and skin cancer. These findings suggest that using AI/NLP for creating research questions is a promising method to advance patient-centered research by addressing patients’ priorities.

Collecting patient perspectives on a large scale is challenging due to the resource-intensive nature of qualitative interviews. Additionally, patient priorities evolve, complicating the identification of important patient-reported concerns when designing new research studies. This AI-enabled NLP pilot study offers a quantitative and repeatable method to identify key patient concerns, thereby bridging patient issues with health research. By analyzing 614 464 unique messages, we could define the top clinical issues, highlighting current knowledge gaps and generating scientifically meaningful and novel research topics informed by patient input.

### Limitations

This study has limitations, including generating 30 research questions in 2 specialties, which may not generalize to other conditions or populations. We focused on clinical issues, excluding administrative support needs. Experts were from a single institution, which may introduce bias. Further studies should include larger samples, diverse specialties, and broader assessors, and consider exploring using representative messages or more recent issues. Evaluating patient perspectives on AI-generated topics and fostering collaboration with investigators and funding agencies is essential for identifying truly important issues.

## Conclusions

This case series demonstrates that an AI/NLP–based framework can systematically prioritize patient concerns to guide patient-centered research despite the inconsistent quality by topic. Overall, we observed that AI-generated research topics could meaningfully guide future health research directions.

## Notes

Supplement 1. **eMethods 1.** More methodological details **eTable 1.** A full list of key topics and keywords that NLP defined after analyzing patient portal messages from those with breast or skin cancer, Stanford Health Care 2013-2024 **eMethods 2.** Engineered prompts used to interpret the key topics from NLP and draft research topics tailored to such topics **eTable 2.** Comparison of keyword-based vs. raw-text-based interpretation of patient issues **eTable 3. **Literature search results and rationales for final decision **eTable 4.** A full list of scores of domain experts for AI-generated research topics **eTable 5.** Demographic characteristics of patients with cancer (breast or skin) in secure messaging of SHC 2013-2024

Supplement 2.
Data Sharing Statement
