# Supplementary material for: Patient-Centered Research Through Artificial Intelligence to Identify Priorities in Cancer Care
Source: JAMA Oncol. 2025 Apr 24;11(6):630–5. doi: 10.1001/jamaoncol.2025.0694 (PMC12022861; doi:10.1001/jamaoncol.2025.0694)
Supplement: Supplement 2. — Data Sharing Statement [file jamaoncol-e250694-s002.pdf]

Data Sharing Statement

Kim. Patient-Centered Research Through Artificial Intelligence to Identify Priorities in Cancer Care. *JAMA Oncol*. Published April 24, 2025. doi:10.1001/jamaoncol.2025.0694

Data
Data available: Yes
Data types: Data (not involving human participants)
How to access data: We have shared the code (https://github.com/JK0902/BERTopic_BIRCH_PCA) for topic modeling that we used to identify the keywords although we are unable to share the original patient message data for privacy protection even with de-identification. the data generated by this research was provided in Supplementary file including the identified keywords, engineered prompts used for AI topic generation.
When available: With publication

Supporting Documents
Document types: Statistical/analytic code
How to access documents: We have uploaded all the code to the public repository along with ReadMe file to guide the potential users (https://github.com/JK0902/BERTopic_BIRCH_PCA)
When available: beginning date: 02-12-2025

Additional Information
Who can access the data: Anyone interested in our study can have access to the code repository.
Types of analyses: For those with research purposes
Mechanisms of data availability: without investigator's support, code will be accessed.
